# Smoking and job satisfaction among laboratory staff

**DOI:** 10.1192/j.eurpsy.2026.10815

**Published:** 2026-07-31

**Authors:** I. Sellami, A. Abbes, A. Feki, D. Elleuch, M. L. Masmoudi, M. Hajjaji, K. Jmal Hammami

**Affiliations:** 1 Occupational Medicine; 2 Rheumatoloy, Hedi Chaker Hospital; 3 Family Department, Medecine University, Sfax, Tunisia

## Abstract

**Introduction:**

Smoking is a common coping behaviour in stressful work environments, including healthcare settings. Exploring its relationship with job satisfaction among laboratory staff may provide insights into occupational well-being.

**Objectives:**

This study aims to assess the prevalence of smoking among laboratory staff and examine its link with job satisfaction.

**Methods:**

We conducted a cross-sectional study of hospital laboratory staff. A self-administered questionnaire was used to collect sociodemographic data, smoking status, degree of nicotine dependence assessed by the Fagerström test, perception of workload, and job satisfaction. The job satisfaction was rated on a 0–10 scale, with 0 representing low and 10 representing high job satisfaction.

**Results:**

A total of 60 participants were included. The mean age was 43.7 ± 9.3 years, with a predominance of females (90%). The married participants represented 76.7% of studied population. The prevalence of smoking was 10%. The Fagerström test showed that 83.3% of smokers had moderate to severe nicotine dependence. The mean perceived job satisfaction was 7.3 ± 1.7. A significant association was observed between nicotine dependence and low job satisfaction (p = 0.03).

**Conclusions:**

Although the prevalence of smoking among laboratory staff was low, the level of nicotine dependence was severe and significantly associated with lower job satisfaction. These findings highlight the need for workplace health programs that address nicotine dependence and promote job satisfaction among laboratory staff.

**Disclosure of Interest:**

None Declared

